# Expression of *TNF*, *IL1B*, and *NOS2* in the neural cell after induced by *Porphyromonas gingivalis* with and without coating antibody anti *-Porphyromonas gingivalis*

**DOI:** 10.12688/f1000research.26749.2

**Published:** 2021-03-17

**Authors:** Endang Winiati Bachtiar, Citra F. Putri, Retno D. Soejoedono, Boy M. Bachtiar

**Affiliations:** 1 Oral Biology and Oral Science Research Center, Faculty of Dentistry Universitas Indonesia, Jakarta, DKI, 10430, Indonesia; 2 Department of Infectious Diseases and Veterinary Public Health, Faculty of Veterinary Medicine, IPB University, Bogor, Indonesia

**Keywords:** Porphyromonas gingivalis, Blocking Antibody, Neuroinflammation, TNF-α, IL-1β, iNOS

## Abstract

*Porphyromonas gingivalis* has virulence factors such as gingipain and lipopolysaccharide, causing bacteremia to reach the brain and activate neuroinflammatory release cytokines. This study analyzed the effect of the co-culture of neuron cells with *P. gingivalis* coated with anti *-P. gingivalis* antibodies against cytokines produced by neuron cells. The gene expressions of the *TNF*, *IL1B*, *NOS2* in neurons was evaluated using RT-qPCR. The results showed that *P. gingivalis* coated with anti *-P. gingivalis* antibody before co-culture with neuron cells could decrease the gene expression of *TNF*, *IL1B*, and *NOS2* of neuron cells.

## Introduction

Periodontitis is an infectious disease that causes inflammation of the tooth-supporting tissue, loss of bone adhesions, initiated by the main pathogens, *Porphyromonas gingivalis*. These bacteria are Gram-negative and have virulence factors such as fimbriae, gingipain, and lipopolysaccharide (LPS), which play a critical role in inducing periodontitis. With this virulence factor, *P. gingivalis* and its products not only damage the periodontal tissue but can also enter the blood circulation or bacteremia and cause systemic spread ^1, 2^. *P. gingivalis* can move to other organs such as the heart and brain. Sophie's research found the presence of LPS *P. gingivalis* in the brains of Alzheimer's patients ^3^. The mechanism for invading *P. gingivalis* bacteria into brain tissue is by penetrating the blood-brain barrier and damaging neuron cells ^4^. When entering the central nervous system, these bacteria will first activate defense cells in the brain, namely the microglia, and astrocytes. Activation of both then releases neuroinflammatory mediators such as TNF-α and IL-1β. Several studies have stated that neuron cells themselves can also release the neuroinflammatory mediators TNF-α and IL-1β triggered by foreign bodies such as bacteria. This excessive release of neuroinflammation is toxic to neuron cells and can cause their damage and death ^5, 6^. Besides, the excessive release of inducible nitric oxide synthase (iNOS) molecule due to antigen by neuron, microglia, and astrocyte cells, may induce human brain neurodegeneration ^7^.

As a form of defense against bacterial attack, the body will naturally produce antibodies to eliminate bacteria. The antibodies produced by the host can specifically recognize certain bacterial species. Either monoclonal or polyclonal antibodies can recognize the lipid A region of the LPS of Gram-negative bacteria, such as *P. gingivalis* ^8^. Animal studies by Barekzi *et al*. stated that pooled human polyclonal antibodies that are injected locally in the area of injury in mice have broad-spectrum antimicrobial effects against Gram-negative bacteria ^9^. *P. gingivalis* reside in a structured community of biofilm attached to surfaces embedded in the extracellular matrix which they produce themselves and they are difficult to eradicate due to their resistance to antimicrobials and the body's defense mechanisms ^10^. The passive immunization approach using polyclonal antibodies to inhibit *P. gingivalis adhesion* to the periodontium tissue is a strategy to prevent biofilm formation and periodontium tissue damage which can lead to deeper tissue invasion so that *P. gingivalis* can enter the systemic circulation. This study aims to evaluate the effect of anti *-P. gingivalis* antibodies on *TNF*, *IL1B*, and *NOS2* gene expression when bacteria interact with neuron cells. We hypothesized that there are differences in the gene expression of *TNF*, *IL1B* and *NOS2* in SHSY-5Y cells that have been exposed to *P. gingivalis* with and without antibody coating.

## Methods

### Cell lines

**Figure 1.**
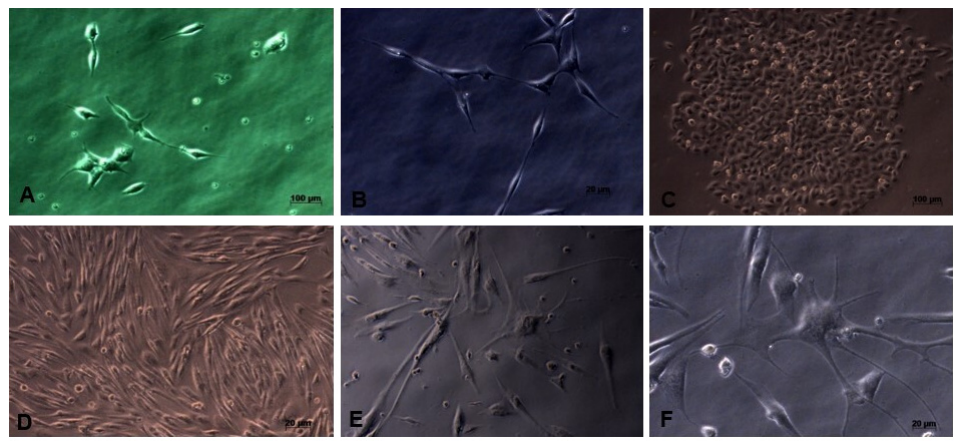
The morphology of the cultured neuron cell line SHSY-5Y; it appears that the SHSY-5Y cells have a neuronal-like cell shape. ( **A**) Cell image of 3 days culture (40x enlargement). ( **B**) Cell image after 7 days, showing elongation of neuron cell bodies and cells growing in groups (40x magnification). ( **C**) There is an increase in cell proliferation and group cell growth (20x magnification). ( **D**) The cells have reached 80% confluence and are ready to be harvested (20x magnification), ( **E** and **F**) SHSY-5Y cells have undergone differentiation, seen the presence of axons from cells and the cell proliferation process begins to decline (40x magnification). *Underlying data* shows raw, unprocessed images used to generate this figure ^12^.

This research is an experimental laboratory study with post test only control group design. This study used the neuron cell line SHSY-5Y (Elabscience, USA), originating from a four-year-old human’s bone marrow neuroblastoma. The cell culture medium was DMEM High Glucose with L-glutamine (Caisson Labs, USA), 15% FBS (Gibco, South America), and 1% Antibiotic-Antimycotic (Gibco, USA). The cultured condition was 5% CO2 at 37°C incubator until 90% confluency was achieved ( Figure 1) ^11^.

*Porphyromonas gingivalis* ATCC 33277 was cultured in Brain Heart Infusion (BHI) agar as a growth medium and incubated under anaerobic conditions with a temperature of 37°C for 24 hours. Then cultured into BHI broth and incubated again under anaerobic conditions with a temperature of 37°C for 24 hours. Then stored at 4°C until ready to use.

This study also used *P. gingivalis* ATCC 33277 bacterial culture. The multiplicity of infection (MOI) used was 1:100, the number of bacteria was 3.6 × 10 ^7^ CFU/mL, and the number of neuron cells was 8 × 10 ^5^ cells/well. In addition, this research used serum anti *-P. gingivalis* antibodies obtained from rabbits after immunization of killed *P. gingivalis. P. gingivalis* antisera were obtained from one-month-old rabbits that have been immunized with 1 mL of 1.7 × 10 ^8 ^CFU/mL *of P. gingivalis* culture. The bacteria were inactivated at 60°C for 30 min before being injected intravenously to the rabbit for 8 weeks with two boosters in intervals of 2 weeks. The animals were euthanized by anesthetic ether inhalation and injection by overdose of anesthetic drug (ketamine 50 mg/kg IM and xylazine 10 mg/kg IM), which caused the animal to fall asleep then slowed and eventually stopped the heart. The blood serum was determined by agar gel precipitation test (AGPT) and the antibody was purified using the Qiagen (QIAGEN, Inc., Valencia, Calif.) protein purification kit, following the manufacturer's protocol. Ethical clearance was given by the Ethical Research Committee of Medical Faculty Universitas Indonesia (2020, number 19-11-1402).

### Coating of anti- *P. gingivalis* antibodies

The antisera coated *P. gingivalis* (3.6 × 10 ^7^ CFU/mL) was prepared by 1:300 diluted rabbit antibody serum in 150 µL growth medium (DMEM High Glucose with L-glutamine (Caisson Labs, USA), 15% FBS (Gibco, South America), and 1% Antibiotic-Antimycotic (Gibco, USA)) for the treatment group; the control was *P. gingivalis* (3.6 × 10 ^7^ CFU/mL) in 150 µL growth medium and the growth medium only without addition of bacteria. The tubes were then incubate for 1 hour in an incubator with a temperature of 37°C ^13^.

### Experimental design

The experiment design as follows: group A was the neurons plus bacteria with antibody coating, and group B for the neuron group plus bacteria without antibody coating and medium only, with 6 replications of each group.

### Harvest of SHSY-5Y neuron cells

Neuron cell cultures that had reached 80% confluence were harvested using 0.25% trypsin-EDTA (Gibco, Canada). The number of cells harvested was counted using a hemocytometer (number of cells 8×10 ^5^ cells/well). The cells were then transferred to a 15 mL tube and resuspended in 2 mL growth medium and then divided into well plates that have been designed with each well containing 100 µL (4×10 ^3^ cells/well) of SHSY5Y cells. The neuron cell line SHSY-5Y (Elabscience, USA) is a cell that has epithelial-like cell and neuronal-like cell morphology and has a cell density of more than 1×10 ^6^ cells/cm ^2^. In this study, cell culture was carried out with two subcultures in January 2020 and February 2020 until the number of cells reached 8×10 ^5^ cells/well. Observation with a microscope was carried out every 2–3 days to identify neuron cells and determine the stage of neuron cell differentiation ( Figure 1)

### *P. gingivalis* exposure to SHSY-5Y cells

Each well of 96 well culture plate filled with SHSY-5Y cells and antibody-coated *P. gingivalis* bacteria and incubated for one hour were added. Group A was filled with 30 µL (1×10 ^5^ CFU/mL) *P. gingivalis* coated with antibody, while group B was filled with 30 µL of bacterial *P. gingivalis* without antibodies. After that, cells were incubated for 24 hours at 37°C. All cells present in the well plate were evaluated for the morphological features, then harvested for RNA extraction.

### RNA extraction and RT-qPCR

The neural cell culture was harvested, and RNA extracted for cDNA synthesis using a Reverse Transcription Kit (ReverTra Ace®, Toyobo, Japan) in line with the manufacturer’s instructions. The pooled cDNA sample is ready for use in the Real-Time PCR tool, with the selected primers as Table 1. RT-PCR was performed using the SYBR Premix Ex Taq TM kit. Relative expression of the target gene normalized to GAPDH, gene expression was analyzed using the 2 ^-ΔΔCt^ method and compared to control. The gene expression of *TNF*, *IL1B* and *NOS2* were evaluated by RT-qPCR as previously reported ^16^

**Table 1.** Primers used in this study.

Primer Name	Sequences	Reference
*TNF*	Forward: 5 'CTG AAC TTC GGG GTG ATC G 3' Reverse: 5 'GCT TGG TGG TTT GCT ACG AC 3'	14
*IL1B*	Forward: 5'-TAT TAC AGT GGC AAT GAG G-3 Reverse: 5'-ATG AAG GGA AAG AAG GTG-3'	14
*NOS2*	Forward: , 5′-GCA GAA TGT GAC CAT CAT GG-3′ Reverse: 5′-ACA ACC TTG GTG TTG AAG GC-3	15
*GAPDH*	Forward: 5'-CTG CAC CAC CAA CTG CTT AG-3’ Reverse: 5'-AGG TCC ACC ACT GAC ACG TT-3'	14

## Results

**Figure 2.**
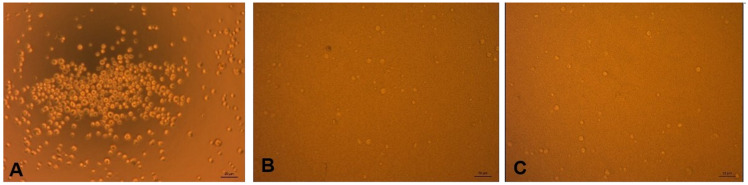
( **A**) SHSY-5Y cell before exposure to *P. gingivalis*, ( **B**) After exposure to *P. gingivalis* without antibodies, ( **C**) After exposure to *P. gingivalis* with antibodies. (Light microscope, 20x magnification).

Figure 2 shows the microscopic morphology of the SHSY-5Y cells that were not exposed to *P. gingivalis*, and those exposed to *P. gingivalis* and coated with anti- *P. gingivalis* antibodies. From these figures, it is known that cells not exposed to *P. gingivalis* grew more than cells exposed to *P. gingivalis*, both with and without antibodies.

**Figure 3.**
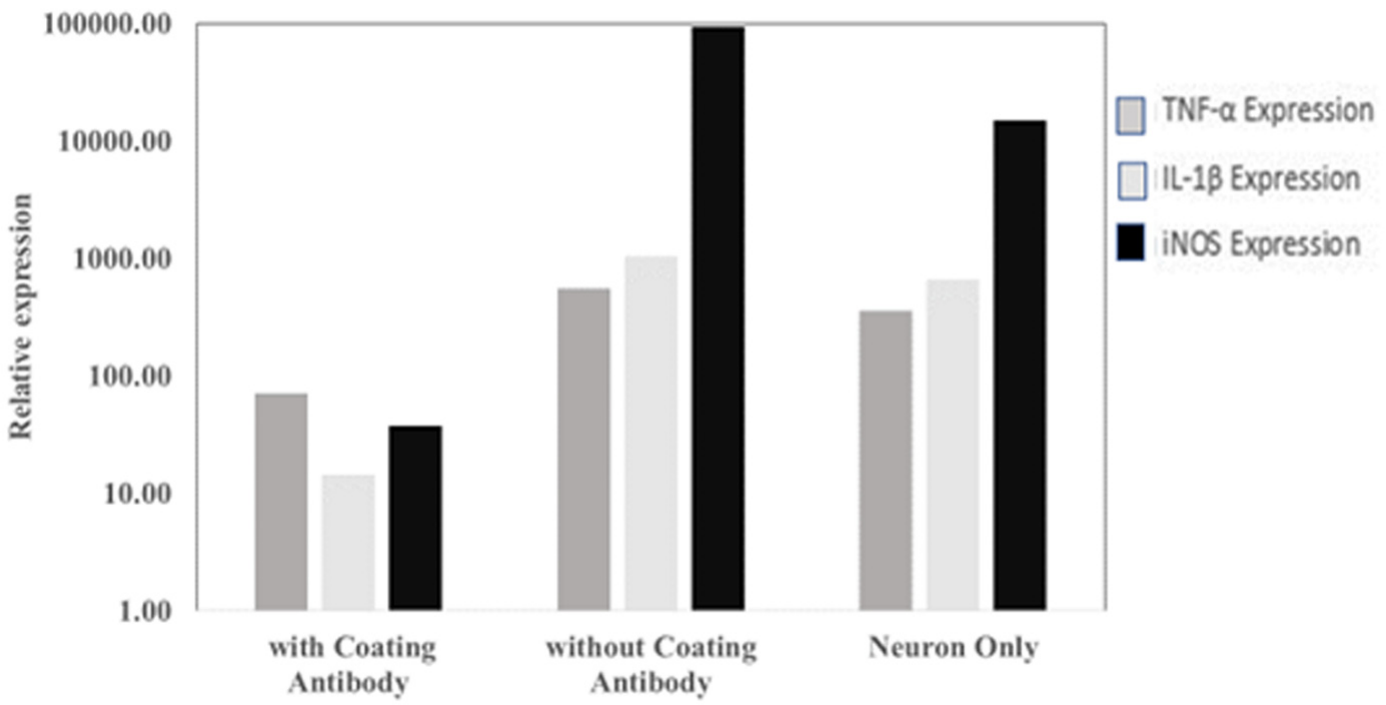
The level of *TNF*, *IL1B and NOS2* gene expression in the antibody-coated group, without antibody coating and neuron cells only.

From qPCR analysis, it was observed that there are differences in the gene expression of TNF-α, IL-1β and iNOS in SHSY-5Y cells that have been exposed to *P. gingivalis* with and without antibody coating. Based on this analysis, it can be concluded that the research hypothesis is accepted. This is shown in Figure 3, where the expression of TNF-α and IL-1β genes in the antibody-coated group was lower than in the antibody-coated group. Ct values are available as *Underlying data* ^17^.

## Discussion

The SHSY-5Y neuron cell line (Elabscience, USA) is a cell derived from human neuroblastoma and taken from bone marrow tissue. These cells have epithelial-like cell and neuronal-like cell morphology. During culture, SHSY-5Y cells can grow into two types of cells, namely adherent cells and floating cells, both of which are viable. However, in this study, adherent cells were used because they were clearer in morphology and proliferation development, and were easy to evaluate after a routine medium change ^18, 19^.

Microscopy images of SHSY-5Y cells ( Figure 1) showed significant growth changes over time. According to Kovalevich and Langford, one of the considerations for the success of SHSY-5Y cell culture is the growth medium used ^19^. In this study, DMEM growth medium containing L-glutamine was used. Glutamine can help increase neuron cell viability and increase neuron cell density, so that it can be seen on microscopy images that neuron cell cultures grow well. However, the number of cells collected until the end of cell culture is 8×10 ^5^, where this number is limited for the study sample. This may occur because cells have started to enter the differentiation stage, so that the cell proliferation process tends to decrease ^18^.

TNF-α and IL-1β are inflammatory mediators released by immune cells when a stimulus triggers the cells. In the nervous system, TNF-α and IL-1β are usually released by astrocytes and microglia cells. However, a number of studies suggest that these inflammatory mediators are also released in large numbers by neuron cells when there are intrinsic or extrinsic triggers ^20^. Extrinsic triggers such as LPS presence from *P. gingivalis* bacteria can trigger the expression of TNF-α and IL-1β by neuron cells so that it can damage neuron cells ^21– 23^. In the incidence of Alzheimer's disease, the release of this inflammatory mediator can cause neuronal cell death, according to a study by Janelsins *et al*., which stated that the inflammatory mediators TNF-α and IL-1β appears to be directly proportional to Alzheimer's disease severity ^23– 25^.

This study is in line with the research of Janelsins *et al*., who found that neuron cells can express TNF-α in brain injury in experimental animals. This is evidenced by the detection of the molecules NeuN and TNF-α in the brain of six-month-old mice. In this study, SHSY-5Y neuron cells can also express TNF-α. In addition, Janelsins *et al*. also found that TNF-α contributed to neuron cell death in the brain with Alzheimer's condition. The signaling mechanism is still unknown, but Janelsins *et al*. stated that there was an increase in the expression of TNFRII and Jun transcript as pro-apoptotic signals mediated by TNF-α ^25, 26^.

The expression of iNOS has been characterized in various cell types as an inflammatory mediator during infection, disease, or tissue damage. INOS is expressed by astrocytes, microglia, and a small portion of endothelial cells in the brain. However, under conditions of increased inflammatory activation in neuron cells, neurons can also express these cytotoxic agents and other reactive oxidative species. The main component that regulates the signaling pathway of iNOS in neurons is the transcription factor NF-κb. The results of this study indicate that anti- *P. gingivalis* antibodies can suppress iNOS expression in neuron cell cultures exposed to *P. gingivalis*. Blocking carried out by antibodies to *P. gingivalis* LPS was thought to suppress bacterial pathogenicity so that iNOS expression in neurons was lower than that of the control group. We assume the antibody use reduced neuronal damage. This is in line with Heneka and Feinstein's research, which states that increased expression of iNOS in neurons can affect neurodegeneration and inflammation in the brain ^7, 27^.

*P. gingivalis* have secreted and non-secreted virulence factors. Secreted virulence factors, for example, gingipain, are virulence factors secreted by bacteria to carry out their activities. Meanwhile, non-secreted virulence factors are virulence factors that are not secreted by bacteria, usually attached to the bacterial structure, such as LPS. In this study, the antibodies used were from injections of killed *P. gingivalis* in rabbits. This will result in the formation of polyclonal antibodies against non-secreted virulence factors, namely LPS, because when it is turned off, the bacteria are unable to secrete other virulence factors such as gingipain. The anti- *P. gingivalis* polyclonal antibodies can recognize *P. gingivalis* bacterial cells and these bacteria's LPS structure ^8, 9, 26^. Therefore, coating this antibody with *P. gingivalis* bacteria for 1 hour before exposure to neuronal cells is thought to block LPS *P. gingivalis* bacteria not to infect neuron cells.

In contrast to the control group that did not use antibodies, *P. gingivalis* was exposed to neuron cells, infecting neuron cells with secreted and non-secreted virulence factors. This occurs because there are no antibodies that block the two types of *P. gingivalis* virulence factors. Therefore, in qPCR analysis results, neuron cell culture with anti *P. gingivalis* antibody showed lower TNF-α and IL-1β expression than the control group. The study ( Figure 3) show that the use of antibodies can suppress the expression of TNF-α and IL-1β. The low expression of TNF-α and IL-1β with the use of antibodies is thought to prevent neuronal damage and is expected to prevent the occurrence of Alzheimer's disease or other cognitive disorders. However, different research results may occur because of the MOI value used. In this study, the MOI used was 1:100.

This study's findings indicate that there is good potential for the development of the anti- *P. gingivalis* vaccine. The anti- *P. gingivalis* antibody used in this study was able to block the development of bacteria *in vitro* so that the neuroinflammatory response can also be minimized. Further research at the *in vivo* level and clinical trials can be developed to see the positive effects of administering antibodies locally or systemically. In the case of local infection of *P. gingivalis* in the oral cavity, the local administration of antibodies may have more potential to suppress bacterial development.

In addition, long-term research involving the role of neuron cells and damage to the central nervous system also needs to be done. With this research, it is hoped that it can become a reference to increase the level of research so that in the future, the prevention of *P. gingivalis* infection can be done so that it can prevent neurodegeneration in the incidence of Alzheimer's disease.

## Conclusion

The cultured SHSY-5Y neuron cells exposed to *P. gingivalis* bacteria after anti- *P. gingivalis* antibody coating exhibited a reduction in the expression of the *TNF*, *IL1B*, and *NOS2*. Further research to see the effectiveness of anti- *P. gingivalis* antibodies still needs to be developed, especially *in vivo*. The success of anti *-P. gingivalis* antibodies in suppressing factors that can damage neuronal cells can be used as a guideline for developing a *P. gingivalis* vaccine, since it is one of the oral bacteria that triggers Alzheimer's disease.

## Data availability

### Underlying data

Open Science Framework: Expression of TNF-α, IL-1β, and iNOS in the neural cell after induced by Porphyromonas gingivalis with and without coating antibody anti-Porphyromonas gingivalis. https://doi.org/10.17605/OSF.IO/Q5CVW ^17^.

This project contains the following underlying data:

- Beta actin GAPDH 2506202_data (1).xls. (qPCR data for housekeeping gene *GAPDH*.)
- IL1b TNFa_data(1).xls. (qPCR data for *IL1B* and *TNF*.)
- iNOS 23062020_data.xls. (qPCR data for *NOS2*.)

Open Science Framework: Expression of TNF, IL1B, and NOS2 in the neural cell after induced by Porphyromonas gingivalis. https://doi.org/10.17605/OSF.IO/JFG3T ^12^.

This project contains the raw images used to produce Figure 1.

Data are available under the terms of the Creative Commons Zero "No rights reserved" data waiver (CC0 1.0 Public domain dedication).
