# Supplementary material for: Expression of TNF, IL1B, and  iNOS2 in the neural cell after induced by Porphyromonas gingivalis with and without coating antibody anti -Porphyromonas gingivalis
Source: F1000Res. 2021 Jun 28;9:1499. Originally published 2020 Dec 23. [Version 4] doi: 10.12688/f1000research.26749.4 (PMC8311800; doi:10.12688/f1000research.26749.4)
Supplement: Peer review history

# Peer review: Reviewer response for version 4

**DOI:** 10.5256/f1000research.58087.r88415

**Published:** 2021-07-20

**Authors:** Hadhimulya Asmara

**Affiliations:** 1 Hotchkiss Brain Institute, University of Calgary, Calgary, AB, Canada

I accept the recent revision.

Is the work clearly and accurately presented and does it cite the current literature?

Yes

If applicable, is the statistical analysis and its interpretation appropriate?

No

Are all the source data underlying the results available to ensure full reproducibility?

Yes

Is the study design appropriate and is the work technically sound?

Yes

Are the conclusions drawn adequately supported by the results?

Partly

Are sufficient details of methods and analysis provided to allow replication by others?

Yes

Reviewer Expertise:

Neuroscience (any works with neurons involvement), Molecular Biology (any works related to DNA and protein).

I confirm that I have read this submission and believe that I have an appropriate level of expertise to confirm that it is of an acceptable scientific standard.

# Peer review: Reviewer response for version 4

**DOI:** 10.5256/f1000research.58087.r88414

**Published:** 2021-06-29

**Authors:** Widya Lestari

**Affiliations:** 1 Department of Oral Biology, Kulliyyah of Dentistry, International Islamic University Malaysia (IIUM), Kuantan, Malaysia

Good and may index as it is.

Is the work clearly and accurately presented and does it cite the current literature?

Yes

If applicable, is the statistical analysis and its interpretation appropriate?

Yes

Are all the source data underlying the results available to ensure full reproducibility?

Yes

Is the study design appropriate and is the work technically sound?

Yes

Are the conclusions drawn adequately supported by the results?

Yes

Are sufficient details of methods and analysis provided to allow replication by others?

Yes

Reviewer Expertise:

Molecular Biologist

I confirm that I have read this submission and believe that I have an appropriate level of expertise to confirm that it is of an acceptable scientific standard.

# Peer review: Reviewer response for version 3

**DOI:** 10.5256/f1000research.56228.r84063

**Published:** 2021-05-06

**Authors:** Hadhimulya Asmara

**Affiliations:** 1 Hotchkiss Brain Institute, University of Calgary, Calgary, AB, Canada

I have questions for the author regarding the amendments from version 2 above. In the last paragraph at the last sentences, the author mentioned "Using 24 well plate cultures will get more appropriate RNA samples to be analyzed in order to overcome this problem and the statistical analysis will be employed."

 My questions are:

1. Will the author change the experiments using 24 well plate in order to overcome the problem of analyzing RNA samples?
2. Will the author employ the statistical analysis as mentioned in the sentences?

I hope the author can clarify my questions above.

Is the work clearly and accurately presented and does it cite the current literature?

Yes

If applicable, is the statistical analysis and its interpretation appropriate?

No

Are all the source data underlying the results available to ensure full reproducibility?

Yes

Is the study design appropriate and is the work technically sound?

Yes

Are the conclusions drawn adequately supported by the results?

Partly

Are sufficient details of methods and analysis provided to allow replication by others?

Yes

Reviewer Expertise:

Neuroscience (any works with neurons involvement), Molecular Biology (any works related to DNA and protein).

I confirm that I have read this submission and believe that I have an appropriate level of expertise to confirm that it is of an acceptable scientific standard, however I have significant reservations, as outlined above.

# Response

**Published:** 2021-06-10

**Authors:** Boy Muchlis Bachtiar

**Affiliations:** Universitas Indonesia, Indonesia

Dear reviewer,

 I would like to clarify the questions related mention: " The limitation to the use of 96 well plate cultures is that the number of cells in each well is very limited. Maybe for future research, using 24 well plate cultures will get a more appropriate RNA sample to be analyzed in order to overcome this problem to be able to use statistical analysis".

 The reviewer's questions are:

1. Will the author change the experiments using 24 well plate in order to overcome the problem of analyzing RNA samples?
2. Will the author employ the statistical analysis as mentioned in the sentences?

The author's response:

 We apologize, maybe the sentence above is confusing. What we mean is for future research the use of well 24 plates. We mean this is a discussion that might be suggested to be applied to future research so that statistical analysis can be applied.

 We hope you understand what we mean and we will be happy to wait for your suggestions if there is any need to improve the manuscript.

 Thanks in advance,

 With warm regards,

 BACHTIAR EW

# Response

**Published:** 2021-06-24

**Authors:** Boy Muchlis Bachtiar

**Affiliations:** Universitas Indonesia, Indonesia

Dear Reviewers,

 Thanks for your valuable suggestions. I would like to inform you that we have revised the manuscript according to your suggestion in 211-213 page as follows: 'The limitation of using 96 well plate cultures is that the cell number in each well is a small amount. Therefore, a future study using 24 well plate cultures is needed to get more appropriate RNA samples to be analyzed'. Thank you in advance. 

 Looking forward to hearing from you.

 With warm regards

 BACHTIAR EW

# Peer review: Reviewer response for version 3

**DOI:** 10.5256/f1000research.56228.r84062

**Published:** 2021-04-29

**Authors:** Widya Lestari

**Affiliations:** 1 Department of Oral Biology, Kulliyyah of Dentistry, International Islamic University Malaysia (IIUM), Kuantan, Malaysia

Approved

Is the work clearly and accurately presented and does it cite the current literature?

Yes

If applicable, is the statistical analysis and its interpretation appropriate?

Yes

Are all the source data underlying the results available to ensure full reproducibility?

Yes

Is the study design appropriate and is the work technically sound?

Yes

Are the conclusions drawn adequately supported by the results?

Yes

Are sufficient details of methods and analysis provided to allow replication by others?

Yes

Reviewer Expertise:

Molecular Biologist

I confirm that I have read this submission and believe that I have an appropriate level of expertise to confirm that it is of an acceptable scientific standard.

# Peer review: Reviewer response for version 2

**DOI:** 10.5256/f1000research.55228.r81562

**Published:** 2021-04-01

**Authors:** Hadhimulya Asmara

**Affiliations:** 1 Hotchkiss Brain Institute, University of Calgary, Calgary, AB, Canada

*Author’s responses:*

*We have deleted ‘morphology’  In Figure 2, it can be seen that there is a decrease in the number of neuron cells after being exposed to P. gingivalis bacteria.*

 Comment:

 Even though the author has already deleted the term “morphology” in figure 2 and in the figure legend, but in the explanation in the result section (first paragraph), the author still states the term “morphology” which is not represented on figure 2.

*Author’s responses:*

*Qualitatively, from the microscope image (Figure 2), there was no difference between the antibody group and the non-antibody group. In our opinion, the number of cells seen on a microscope does not necessarily indicate the expression of neuroinflammation. Based on the results of the real time PCR analysis, it showed that the expression of neuroinflammation was more in the group without antibodies (Figure 3).*

 Comment:

 The author’s response does not address the concern of the result in figure 2 that it is against or at least does not support the author’s hypothesis. The number of cell growth in the cells that are treated with antibody coated are supposed to be higher than the one without the antibody coated. Yet there is no explanation from the author about this data in the result and discussion sections in correlation with the hypothesis. In my opinion, the author should explain the result of this figure 2 data, why does not this result support the hypothesis and why does the cell numbers growth does not differ between coating and without coating.

*Author’s responses:*

*We used pooled samples (we have added this information in the methods section)  as there was insufficient amount of RNA from each individual replication of the experiments. But we think the value of gene expression presented here is equal to de average of 6 replications of experiment. Hence, we couldn’t get a statistical significance. Some studies also use this kind of data interpretation (Shu-Dong Zhang, Timothy W. Gant, Effect of pooling samples on the efficiency of comparative studies using microarrays, Bioinformatics, Volume 21, Issue 24, 15 December 2005, Pages 4378–4383)*

 Comment:

 The author cannot show the statistical significance or P values of the different expressions of these genes instead using pooled samples to justify the different values of the gene expression. There is a report about the weaknesses of using pooled samples method. According to that report, the pooled samples method has some biases since the equal amount of RNA was used for each individual sample are not the same and it may cause some alteration of individual RNA contributions such that some samples dominate more than others in the pooled expression. Another disadvantage is that one may not be able to associate the gene expression from the pooled sample with the individual phenotypic information, and thus cannot make certain statistical inference or predictions for individuals. Based on those disadvantages, the authors of that report suggested everyone has to be cautious about designing a pooled experiment. They also suggested if there is not enough RNA from each individual sample to run an array, the number of different pools should not be too small and the number of subjects should be appropriately increased to compensate for the loss of degrees of freedom and decrease in power caused by pooling samples (Shih, J.H., et. al., 2004). ^1^ If the author of this paper can address those disadvantages and follow suggestions in the paper referred, the different value of gene expression might be justifiable.

 I would like the author to address all my concerns above appropriately, until then, in my opinion, this paper is not yet ready for indexing.

Is the work clearly and accurately presented and does it cite the current literature?

Yes

If applicable, is the statistical analysis and its interpretation appropriate?

No

Are all the source data underlying the results available to ensure full reproducibility?

Yes

Is the study design appropriate and is the work technically sound?

Yes

Are the conclusions drawn adequately supported by the results?

Partly

Are sufficient details of methods and analysis provided to allow replication by others?

Yes

Reviewer Expertise:

Neuroscience (any works with neurons involvement), Molecular Biology (any works related to DNA and protein).

I confirm that I have read this submission and believe that I have an appropriate level of expertise to confirm that it is of an acceptable scientific standard, however I have significant reservations, as outlined above.

## References

[1] : Effects of pooling mRNA in microarray class comparisons. *Bioinformatics* .2004;20(18) : 10.1093/bioinformatics/bth391 3318-25 10.1093/bioinformatics/bth391 15247103

# Peer review: Reviewer response for version 1

**DOI:** 10.5256/f1000research.29536.r76420

**Published:** 2021-04-16

**Authors:** Widya Lestari

**Affiliations:** 1 Department of Oral Biology, Kulliyyah of Dentistry, International Islamic University Malaysia (IIUM), Kuantan, Malaysia

This report provides new insight into dentistry. *P. gingivalis *is not only related to Periodontitis but also express in neuron cells, which may give additional knowledge to clinicians. Pathways of expression of TNF alpha and IL-1B area clearly explained. Methodology and results are well established and, we believe these findings will benefit clinicians and also researchers as well.

**The methodology:**

 This is an in vitro study using a nerve cell culture. The experimental design has used a treatment group and a control group which, in my opinion, is good enough to observe the effect of administering anti- *P. gingivalis* antibodies on the expression of neuroinflammatory cytokines.

**The results:**

 The results have been presented clearly.  I suggest analyzing them descriptively without using statistics. This is a preliminary study, in my opinion, it has described how the expression of neuroinflammatory cytokines in nerve cell cultures after exposure to *P. gingivalis *which has been treated with anti- *P. gingivalis *antibody.

 Overall, a minor revision is needed.

Is the work clearly and accurately presented and does it cite the current literature?

Yes

If applicable, is the statistical analysis and its interpretation appropriate?

Yes

Are all the source data underlying the results available to ensure full reproducibility?

Yes

Is the study design appropriate and is the work technically sound?

Yes

Are the conclusions drawn adequately supported by the results?

Yes

Are sufficient details of methods and analysis provided to allow replication by others?

Yes

Reviewer Expertise:

Molecular Biology

I confirm that I have read this submission and believe that I have an appropriate level of expertise to confirm that it is of an acceptable scientific standard.

# Peer review: Reviewer response for version 1

**DOI:** 10.5256/f1000research.29536.r76422

**Published:** 2021-01-25

**Authors:** Hadhimulya Asmara

**Affiliations:** 1 Hotchkiss Brain Institute, University of Calgary, Calgary, AB, Canada

The manuscript by Bachtiar *et al. *presents some potentially interesting findings on preventing the neurons from damage and death by reducing excessive release of neuroinflammatory mediators such as TNF-α and IL-1β and other molecules such as iNOS. This paper highlights the potential success of using antibody specially anti- *P. gingivalis* antibodies for suppressing factors that can damage neuronal cells can be used as a guideline for developing a *P. gingivalis* vaccine since it is one of the oral bacteria that triggers Alzheimer's disease.

 Overall, the work presents an interesting idea regarding possible antibody use to decrease the release of neuroinflammatory agents and other molecules that are toxins and cause harm to the neurons. If this finding is rigorously proven, it could be a great contribution to the prevention and treatment of bacterial infection in Neurodegenerative disease. At this stage, however, there are several comments that need to be clarified in the manuscript that substantively influences the significance of the findings.

**Specific comments:**

1. In the paper, the authors did not mention the efficacy of other treatments such as antibiotic treatment for *P. gingivalis* infection and its effect on the release of neuroinflammatory agents. It is important to highlight the antibiotic’s effect since if the antibiotic is effective enough to kill the bacteria and prevent the infection on neurons then this paper must add more reasons why this paper or the antibody approach is better than the antibiotic treatment.
2. In the figure 2. The authors used the word “morphology” of the neurons, but the authors did not explain the morphology aspect of the neurons (such as elongation axons or shape of the cell body, etc). It only described the change of the growth or number of the viable cells that was decreased by the exposure of *P. gingivalis* with or without antibody coating. It is better to describe how the exact morphological changes of the neurons as clearly shown in figure 1 before the exposure *P. gingivalis*. Even better if the authors can add the qualitative and quantitative analysis of those differences between the groups.
3. The correlation between figure 2 and figure 3. The reducing number of viable cells in figure 2 and the reducing the expression of TNF-α, IL-1β, and iNOS in figure 3 seems does not fit with the hypothesis in the paper. If the expression of TNF-α, IL-1β, and iNOS were decreased on the neurons that were exposed by coating antibody *P. gingivalis *compared to the ones without coating (figure 3) then the number of viable neurons in the figure 2c (expose with antibody) must be higher than figure 2b (without antibody) since the antibody will decrease TNF-α, IL-1β, and iNOS and it means to prevent the neuronal damage and death on figure 2b. What is the clarification or the explanation of this confusion?  
4. It is necessary to state the *P* values or statistical significance of the differences between the three groups in figure 3 (with coating antibody, without coating anti, and neuron only). It is important to conclude that one group is higher or lower than the others groups based on statistical significance or *P* values.
5. As a minor comment, I think there is a typo on the first line of the second paragraph in the discussion section. In my opinion, I think the authors want to show figure 1 instead of figure 2 (as stated in that line) for describing the growth changes of neurons over time.

Is the work clearly and accurately presented and does it cite the current literature?

Yes

If applicable, is the statistical analysis and its interpretation appropriate?

No

Are all the source data underlying the results available to ensure full reproducibility?

Yes

Is the study design appropriate and is the work technically sound?

Yes

Are the conclusions drawn adequately supported by the results?

Partly

Are sufficient details of methods and analysis provided to allow replication by others?

Yes

Reviewer Expertise:

Neuroscience (any works with neurons involvement), Molecular Biology (any works related to DNA and protein).

I confirm that I have read this submission and believe that I have an appropriate level of expertise to confirm that it is of an acceptable scientific standard, however I have significant reservations, as outlined above.

# Response

**Published:** 2021-03-03

**Authors:** Boy Muchlis Bachtiar

**Affiliations:** Universitas Indonesia, Indonesia

Dear Reviewer,

 Thank you for the valuables suggestion in improving of the quality our manuscript.

 Here we would like to respond your feedback. 

**Specific comments:**

1. In the paper, the authors did not mention the efficacy of other treatments such as antibiotic treatment for *P. gingivalis* infection and its effect on the release of neuroinflammatory agents. It is important to highlight the antibiotic’s effect since if the antibiotic is effective enough to kill the bacteria and prevent the infection on neurons then this paper must add more reasons why this paper or the antibody approach is better than the antibiotic treatment.

Author’s responses:

 We thank for this valuable comments. We have inserted the argumentation in the manuscript of this suggestion as follow:

*P gingivalis* reside in a structured community of biofilm attached to surfaces embedded in the extracellular matrix which they produce themselves and they are difficult to eradicate due to their resistance to antimicrobials and the body's defense mechanisms ^10^. The passive immunization approach using polyclonal antibodies to inhibit *P gingivalis* adhesion to the periodontium tissue is a strategy to prevent biofilm formation and periodontium tissue damage which can lead to deeper tissue invasion so that *P gingivalis* can enter the systemic circulation.

  

1. In the figure 2. The authors used the word “morphology” of the neurons, but the authors did not explain the morphology aspect of the neurons (such as elongation axons or shape of the cell body, etc). It only described the change of the growth or number of the viable cells that was decreased by the exposure of *P. gingivalis* with or without antibody coating. It is better to describe how the exact morphological changes of the neurons as clearly shown in figure 1 before the exposure *P. gingivalis*. Even better if the authors can add the qualitative and quantitative analysis of those differences between the groups.

Author’s responses:

 We have deleted ‘morphology’  In Figure 2, it can be seen that there is a decrease in the number of neuron cells after being exposed to *P. gingivalis* bacteria.

1. The correlation between figure 2 and figure 3. The reducing number of viable cells in figure 2 and the reducing the expression of TNF-α, IL-1β, and iNOS in figure 3 seems does not fit with the hypothesis in the paper. If the expression of TNF-α, IL-1β, and iNOS were decreased on the neurons that were exposed by coating antibody *P. gingivalis *compared to the ones without coating (figure 3) then the number of viable neurons in the figure 2c (expose with antibody) must be higher than figure 2b (without antibody) since the antibody will decrease TNF-α, IL-1β, and iNOS and it means to prevent the neuronal damage and death on figure 2b. What is the clarification or the explanation of this confusion?

Author’s responses:

 Qualitatively, from the microscope image (Figure 2), there was no difference between the antibody group and the non-antibody group. In our opinion, the number of cells seen on a microscope does not necessarily indicate the expression of neuroinflammation. Based on the results of the real time PCR analysis, it showed that the expression of neuroinflammation was more in the group without antibodies (Figure 3).

1. It is necessary to state the *P* values or statistical significance of the differences between the three groups in figure 3 (with coating antibody, without coating anti, and neuron only). It is important to conclude that one group is higher or lower than the others groups based on statistical significance or *P* values.

Author’s responses: 

 We used pooled samples (we have added this information in the methods section)  as there was insufficient amount of RNA from each individual replication of the experiments. But we think the value of gene expression presented here is equal to de average of 6 replications of experiment. Hence, we couldn’t get a statistical significance. Some studies also use this kind of data interpretation (Shu-Dong Zhang, Timothy W. Gant, Effect of pooling samples on the efficiency of comparative studies using microarrays, Bioinformatics, Volume 21, Issue 24, 15 December 2005, Pages 4378–4383)

1. As a minor comment, I think there is a typo on the first line of the second paragraph in the discussion section. In my opinion, I think the authors want to show figure 1 instead of figure 2 (as stated in that line) for describing the growth changes of neurons over time.

Author’s responses *:*

 Thank you, we have fixed this typo.

 Again thanks

 With warm regards

 Bachtiar EW
